# Association of Subway Driver's Depressive Symptoms and Experience of Work-Related Problems

**DOI:** 10.4178/epih/e2010010

**Published:** 2010-12-03

**Authors:** Sun-Jin Jo, Hyeon Woo Yim, Hyoung-Ryoul Kim, Kang Sook Lee, Jong-Ik Park, Sung Man Chang

**Affiliations:** 1 Department of Preventive Medicine, College of Medicine, The Catholic University of Korea, Seoul, Korea.; 2 Clinical Research Center for Depression, Seoul, Korea.; 3 Department of Occupational and Environmental Medicine, The Catholic University of Korea, St. Mary's Hospital, Seoul, Korea.; 4 Department of Psychiatry, School of Medicine, Kangwon National University, Chuncheon, Korea.; 5 Department of Psychiatry, School of Medicine, Kyungpook National University, Daegu, Korea.

**Keywords:** Subway, Accident, Depression

## Abstract

**OBJECTIVES:**

Subway drivers experience various types of work-related problems during their driving, and those experiences can act as risk factors for depressive symptoms. This study was conducted to investigate the association between work-related problems and subway driver's depressive symptoms.

**METHODS:**

We recruited all of the 961 current subway drivers of a subway company located in Seoul, South Korea and conducted a survey of their socio-demographic and vocational characteristics, hospital visits as an outpatient or inpatient, and work-related problem experiences during the last year. Work-related problems included an accident resulting in death or injury, a conflict with a customer, a sudden stop from an emergency bell, or a near accident. Depressive symptoms were measured with the Center for Epidemiologic Studies-Depression (CES-D) instrument. The survey was performed using a self-report questionnaire from April 16 to July 13, 2007. The data of 827 drivers (86.2%) were analyzed.

**RESULTS:**

Experience of a conflict with a passenger (p=0.011), a sudden stop from an emergency bell (p=0.001), or a near accident (p=0.001) increased the prevalence of depressive symptoms among subway drivers. A multiple logistic regression analysis revealed that a sudden stop from an emergency bell increased the risk of depressive symptoms significantly (OR=2.59, p=0.026). Near accidents were marginally associated with a higher risk for depressive symptoms (OR=1.62, p=0.062).

**CONCLUSION:**

The experience of a sudden stop from an emergency bell increased subway driver's depressive symptoms, and near accidents may increase the risk of depressive symptoms. Therefore, interventions for the drivers who had experienced these work-related problems are needed.

## INTRODUCTION

While workers' physical health-related issues have mainly been focused on in the past, their mental health has currently become a social issue. In particular, as the occurrence of depression following excessive work stress and the suicide caused by deterioration of depression increases, more attention has been given to the depression of workers.

Depression is commonly observed at the working ages of 18-64 yr. According to a study investigating the prevalence of mental diseases of Koreans aged 18 to 64, the lifetime prevalence of major depressive disorder was 5.6% [1]. Although there has been no data on this subject for actual workers, the healthy worker effect is considered to reduce the prevalence of depression. However, the social burden caused by depression is very serious and the WHO reported that it was one of major diseases leading to physical, social and vocational disorders and this social burden is growing [2].

In England, stress or depression caused or deteriorated by work was found to be the second most common work-related disease following muscular-skeletal diseases [3]. In addition, depression was reported to account for one third of all work-related disease in England, it was the second major cause of long absence and it caused 20% of the early retirement [4]. Every year 15-30% of workers experience mental problems and 20% of all sick leave is explained by a single cause, namely depression [5]. Depression is surely one of the very critical health problems for workers.

In particular, for subway drivers any problem with their mental health is not only a personal problem that damages their own health condition, but it also can be a risk to others. In other words, the mental health of subway drivers is directly related with the safety of many passengers using subways, so it is different from that of other workers. When a subway driver shows a lack of concentration or bad judgment as depressive symptoms, his or her driving or mechanical operation can be inappropriate [5]. Therefore, managing depression of subway drivers is an issue that needs attention.

Twenty to thirty percent of the drivers experience accidents resulting in death or injury [6, 7], and particularly fatal accidents increase the possibility of post-traumatic stress disorder and depressive symptoms because of an excessive guilty conscience even though the accidents occur under unexpected circumstances and they are not related with the driving ability of drivers [7, 8]. Moreover, subway drivers experience various work-related problems such as a conflict with a customer and near accidents during their driving and these can work as important risk factors for depression or as stressors. Kenner et al suggested that relatively minor stressors or "hassles" that characterized every day life are significant for predicting health outcomes [9], and depressed individuals scored higher than the controls on measures of "hassles" [10]. The frequency and intensity of "hassles" are related to more severe depression [11].

Like this, subway drivers are exposed to risk factors for depression, but there has been no research on the prevalence of Korean subway driver's depressive symptoms and there have been no studies on how to manage their depressive symptoms.

Therefore, this study was conducted to investigate the association between depressive symptoms and subway drivers' work-related problems that they experienced during their driving.

## METHODS

### Subjects

All of 961 current subway drivers working for the "A" subway located in Seoul at January 1, 2007 were chosen as the subjects of this study.

### Questionnaire

The questionnaire used for this study included six questions about socio-demographic and vocational characteristics, four questions about work-related problems and hospital visit as an outpatient or inpatient, and twenty questions for the screening of depressive symptoms.

As the socio-demographic and vocational characteristics, gender, age, the education level, the marital status, the length of time working as a subway driver and the status at work were investigated. As work-related problems, an accident resulting in death or injury, a conflict with a customer such as blame from a passenger or a dispute/scuffle with a passenger, a sudden stop due to the emergency bell and a near accident during the last one year were examined. These four self-administrating questions were developed as a result of qualitative interviews with five subway drivers.

The Center for Epidemiologic Studies-Depression (CES-D) Scale that was standardized by Cho and Kim [12] was used to detect depressive symptoms. The CES-D is a self-report scale consisting of 20 questions, and it has been used as a measure of depressive symptoms in epidemiologic studies. It found that the CES-D was useful to detect clinical depression [13]. All the questions of the scale are scored from 0 to 3 points and a person recording 21 or more points out of a total of 60 was classified into the depressive symptom group. It was suggested that the score 21 was appropriate cut-off point for screening depression or depressive symptoms in community epidemiological studies by Cho and Kim [12].

### Data collection

The data was collected from six offices for around two weeks at each office from 16 April to 13 July, 2007. Two trained investigators were dispatched to each office and the subway drivers of each office were individually asked to fill out the questionnaires after informed written consent was obtained. The subjects' confidentiality was assured and the investigation was conducted by using the self-reporting questionnaire. The data from 827 subjects out of 961 was collected and the rate of filling out the questionnaire was 86.2%.

### Data analysis

After dual entry of the collected data was performed by two trained persons, the entry errors were found and corrected and the missing values were replaced by using the nearest neighbor imputation.

χ^2^ tests were used to investigate the difference in the existence of depressive symptoms according to socio-demographic and vocational characteristics and experiencing different types of problems. To determine the effect of each work-related problems on depressive symptoms, multiple logistic regression analysis was performed after controlling for the socio-demographic and vocational characteristics and hospital visit as an outpatient or inpatient.

This study was approved by the Institutional Review Board (IRB) of The Catholic University of Korea.

## RESULTS

The prevalence of depressive symptoms of the subjects was 9.2%. There was no difference in the prevalence of depressive symptoms according to gender, the marital status, the length of time working as a driver, the status at work and the hospital visit as an inpatient. However, the differences according to hospital visit as an outpatient and the education period were statistically significant (p=0.003) or marginally significant (p=0.056). The prevalence of depressive symptoms of the subjects who were educated for less than 16 yr and 16 yr or more were 7.2% and 11.1%, respectively, and the prevalence of subjects with a longer education period was found to be slightly higher than that of the others workers. The subjects who experienced hospital visit as an outpatient showed a higher prevalence of depressive symptoms (12.2%) compared with that of the inpatient (6.5%) (Table 1).

The results of the analysis on the prevalence according to experiencing each work-related problem are presented in Table 2. No difference was observed in the prevalence of depressive symptoms according to experiencing an accident resulting in death or injury for the last one year. For a conflict with a customer, the prevalence of depressive symptoms not experiencing conflict with a customer was 7.1%, while that of the drivers experiencing conflict with a customer was 12.3% (p=0.011). Out of the subjects who had not stopped a subway due to ringing the emergency bell by a passenger, 3.4% showed depressive symptoms and among those having stopped the subway due to the emergency bell of a passenger, 11.1% experienced depressive symptoms (p=0.001). The prevalence of the drivers without and with the experience of a near accident was 7.0% and 14.0%, respectively, and the prevalence of depressive symptoms for the subjects who experienced a near accident was higher than that of the others.

To determine the association between experiencing work-related problems and depressive symptoms among the subway drivers, multiple logistic regression analysis was conducted with the four work-related problems as independent variables and the education level and a hospital visit as an outpatient and gender, which has been reported to be closely related with depressive symptoms, as the control variables. According to the results, a sudden stop due to an emergency bell and a near accident among the four work-related problems significantly increased the prevalence of depressive symptoms. The subway drivers having experienced a sudden stop from an emergency bell were 2.59 times more likely to have depressive symptoms than that of the other drivers (95% CI: 1.08-2.76). Those having experienced near accidents had a 1.67 times greater possibility of experiencing depressive symptoms than that of the other drivers (95% CI: 0.98-2.70) and this was marginally significant. Experience of an accident resulting in death or injury, or a conflict with a customer during the last one year was revealed not to significantly affect the possibility of experiencing depressive symptoms (Table 3).

## DISCUSSION

For making the diagnosis of major depression, at least a two-week continuance of a depressive mood or loss of interest is a necessary condition and five or more symptoms among decreased or increased appetite, insomnia or hypersomnia, psychomotor agitation or retardation, fatigue or loss of energy, feelings of worthlessness, reduced thinking power and repeated thoughts on death should be associated. In particular, major depression can be diagnosed when these symptoms provoke social and vocational dysfunction [14]. Although the Composite International Diagnostic Interview (CIDI) and Diagnostic Interview Schedule (DIS) are mainly used to examine the prevalence of major depression in research on the general population, the time and effort to use them are considerable. Therefore, prevalence of depressive symptoms is generally investigated in epidemiologic studies, except for nationwide surveys. Among the scales for that, the CES-D is a self-reporting depression-screening tool that is most widely utilized around the world and it is known to be appropriate for epidemiologic studies in the community because its questions are very simple and the degree of a depressive symptom is measured along with the time period of the symptoms [15]. This study also used the CES-D to survey the prevalence of depressive symptoms of the subjects.

According to the results, 9.2% of the subjects were found to have depressive symptoms and this level was much lower than that of a general population that was assessed using the CES-D [16]. This was considered to be caused by the health worker effect, as mentioned before. Healthy workers are characteristically hired and their employment provides a relatively improved socioeconomic status, so their health condition is better than that of a general population [17].

This study revealed that experiencing an accident resulting in death or injury during the last one year was not related with subway drivers' depressive symptoms. As we asked about experiencing fatal accidents during the last one year, there was the possibility that any acute depressive symptoms right after accident had disappeared at that time before the survey was performed. When Tranah and Farmer compared the mean scores of the severe depression sub-scale of the General Health Questionnaire (GHQ) at one and six months after exposure to railway suicide, the scores at one month were low, so a marked decrease was not possible at six months [18]. In addition, the CES-D, which was used to measure depressive symptoms in this study, is a tool that does not measure post-traumatic stress disorder, but rather, it measures general depressive symptoms. Therefore, the association between less traumatic work-related problems and depressive symptoms might be revealed.

Subway drivers experience various work-related problems during their driving and a problem leading to the most serious trauma is an accident in which a passenger runs into the railway to commit suicide. This results in a fatal outcome in most cases and a subway driver can not expect it or take measures to prevent it [8]. A driver can see the eyes of the person just before they are killed at a certain place and sometimes he or she deals with that place and drives there again even after the accident [19]. A subway driver with such an experience has a high risk of post-traumatic stress disorder and depression [20]. In Denmark, the necessity of preparation and treatment for such a situation has been recognized and measures to cope with it have been instituted politically. The measures for it include 1) psychotherapy (crisis intervention) within 24 hr of the suicide attempt, 2) preparation for psychological crisis reactions in young drivers during their training and introduction of psychotherapy after the experience of railway suicide, 3) introduction to crisis intervention and psychological first aid for instructors and other staff who are called out in connection with suicide events, and 4) information campaigns inside and outside the company concerning railway suicides [21]. When these measures that were taken in Denmark also happen for the Korean subway drivers and the drivers fully recognize that they are not personally responsible for an accident resulting in death or injury, the effect of such an event on depressive symptoms can be reduced.

On the contrary, a near accident or a sudden stop from an emergency bell of a passenger was observed to significantly or marginally increase the risk of depressive symptoms in this study. This finding is consistent with Tranah and Farmer's report that those symptoms still reported at 6 months were most likely precipitated by the index incident and not by other negative life events that occurred between one month and six months after a fatal accident [18]. A near accident means a situation in which an error of a worker or a defect on an actual spot can provoke an accident, but fortunately a direct accident does not occur. Heinrich suggested a principle of 1:29:300 that out of a total of 330 accidents, the cases of near, minor and serious accidents were 300, 29 and 1, respectively, according to an analysis of around 75,000 accidents [22]. Like this, a near accident is one of the potential stressors of subway drivers. Although an accident resulting in death or injury leads to the biggest psychological reaction of the drivers, a near accident has also been reported to provoke a strong reaction [7]. A near accident is a major cause of stress by itself, and for subway drivers who have experienced an accident resulting in death or injury, a near accident or a sudden stop due to an emergency bell can produce a strong emotional impact on them by reminding them of the past accident.

Gender has been reported as an important risk factor for the prevalence of depression. According to a survey on the one-year prevalence of depression conducted in the U.S. using the Composite International Diagnostic Interview, the prevalence of depression of males and females was 7.7% and 12.9%, respectively [23]. In Korea lifetime prevalence of depression for females was also higher than that of males at 7.6% and 3.6%, respectively [1] and the same trend was observed in this study. However, the subjects included only thirteen females and there existed some possibility of chance in the prevalence of depressive symptoms among the females.

Whether a low educational background is a risk factor for depression is still controversial [24-26]. While a high educational background in males is associated with a low prevalence of depression, in females this tendency has not been clearly observed [1, 26]. In this study in which the subjects were mostly males, a higher level of education was associated with a higher possibility of experiencing depressive symptoms. When the level of education is investigated as a variable of the socio-demographic characteristics, an education level of 12 or less than 12 yr is generally used as a dividing line and more than 12 yr is generally defined as a long education period. However, because all of the subjects of this study had a high level of education of more than 12 yr, the period was divided into less than 16 yr and 16 or more years to analyze its association with depressive symptoms. As a result of this study, a higher level of education showed a higher prevalence of depressive symptoms compared to that of a shorter education period, and this might be related with organizational culture. When qualitative interviews were done with the subway drivers of this company, it found that there existed some delicate conflicts between the drivers who graduated from a particular vocational high school/college and other schools. The analysis of the relation between the level of education and the status at work resulted in significant negative correlation. In light of these results, it is possible that the subway drivers having a higher level of education were not included in the alumni group of a particular high school or college and who have vested interests in this company and who have felt some conflicts and revealed depressive symptoms.

Depression of subway drivers should be recognized as an important issue in terms of safety of citizen as well as for the driver's personal health. Among the various problems experienced by subway drivers during their driving, a sudden stop due to an emergency bell and a near accident were found to have a high possibility of working as risk factors for depressive symptoms. Therefore, interventions related to not only to accidents resulting in death or injury, but also to those problems should be done.

This study was performed as a cross-sectional design study; therefore, there exist some limitation to explain the temporal relationship between work-related problems and depressive symptoms. Generalization of the results of this study should be done with caution as most of the subjects were males.

## Figures and Tables

**Table 1:**
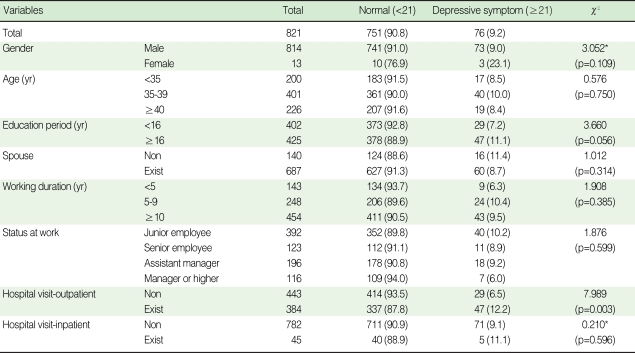
Prevalence of depressive symptoms according to the socio-demographic and vocational characteristics and hospital visit as an outpatient or inpatient

^*^Fisher's exact test.

**Table 2:**
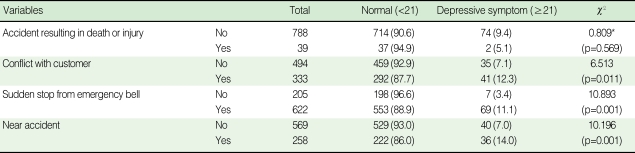
Prevalence of depressive symptoms according to work-relate problems during the past year

^*^Fisher's exact test.

**Table 3:**
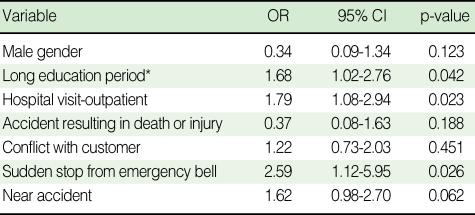
Results of multiple logistic regression analysis to explore the risk factor

Reference group: less than 16 yr. OR, odds ratio; CI, confidence interval.

## References

[1] Cho MJ, Chang SM, Cheon HS, Lee HJ, Kim SK, Lee YR (2007). The epidemilogical survey of psychiatric illnesses in Korea 2006.

[2] World Health Organization (2001). Burden of mental and behavioral disorders, in World Health Report.

[3] Teasdale EL, Creed FH, Baxter PJ, Adams PH, Aw TC, Cockcroft A, Harrington JM (2000). Health and mental illness at work: clinical assessment and management. Hunter's disease of occupations.

[4] Glozier N (2002). Mental ill health and fitness for work. Occup Environ Med.

[5] Kearns J, Lipsedge M, Cox R, Edward F, Palmer K (2000). Psychiatric disorders. Fitness for work: the medical aspect.

[6] Park SY, Kim HL, Yim HW, Jo SJ, Lee GS (2007). Medical utilization and general health status according to experience of accidents among subway driver. Book of abstract of 39th Conference.

[7] Vatshelle A, Moen BE (1997). Serious on-the-track accidents experienced by train drivers: psychological reactions and long-term health effects. J Psychosom Res.

[8] Cothereau C, de Beaurepaire C, Payan C, Cambou JP, Rouillon F, Conso F (2004). Professional and medical outcomes for French train drivers after "person under train" accidents: three year follow up study. Occup Environ Med.

[9] Kanner AD, Coyne JC, Schaefer C, Lazarus RS (1981). Comparison of two modes of stress measurement: daily hassles and uplifts versus major life events. J Behav Med.

[10] Ravindran AV, Matheson K, Griffiths J, Merali Z, Anisman H (2002). Stress, coping, uplifts, and quality of life in subtypes of depression: a conceptual frame and emerging data. J Affect Disord.

[11] Klein DN, Lewinsohn PM, Seeley JR (1997). Psychosocial characteristics of adolescents with a past history of dysthymic disorder: comparison with adolescents with past histories of major depressive and non-affective disorders, and never mentally ill controls. J Affect Disord.

[12] Cho MJ, Kim KH (1993). Diagnostic validity of the CES-D (Korean version) in the assessment of DSM-III-R major depression. J Korean Neuropsychiatr Assoc.

[13] Weissman MM, Sholomskas D, Pottenger M, Prusoff BA, Locke BZ (1977). Assessing depressive symptoms in five psychiatric populations: a validation study. Am J Epidemiol.

[14] American Psychiatric Association (1994). Diagnostic and statistical manual of mental disorders.

[15] Roberts RE, Vernon SW (1983). The Center for Epidemiologic Studies Depression Scale: its use in a community sample. Am J Psychiatry.

[16] Cho MJ, Kim KH (1998). Use of the Center for Epidemiologic Studies Depression (CES-D) Scale in Korea. J Nerv Ment Dis.

[17] Wen CP, Tsai SP (1982). Anatomy of the health worker effect - a critique of summary statistics employed in occupational epidemiology. Scand J Work Environ Health.

[18] Tranah T, Farmer RD (1994). Psychological reactions of drivers to railway suicide. Soc Sci Med.

[19] Williams C, Miller J, Watson G, Hunt N (1994). A strategy for trauma debriefing after railway suicides. Soc Sci Med.

[20] Malt UF, Karlehagen S, Hoff H, Herrstromer U, Hildingson K, Tibell E (1993). The effect of major railway accidents on the psychological health of train drivers--I. Acute psychological responses to accident. J Psychosom Res.

[21] Tang D (1994). Psychotherapy for train drivers after railway suicide. Soc Sci Med.

[22] Heinrich HW (1980). Industrial accident prevention.

[23] Kessler RC, McGonagle KA, Zhao S, Nelson CB, Hughes M, Eshleman S (1994). Lifetime and 12-month prevalence of DSM-III-R psychiatric disorders in the United States. Results from the National Comorbidity Survey. Arch Gen Psychiatry.

[24] Kessler RC, Zhao S, Blazer DG, Swartz M (1997). Prevalence, correlates, and course of minor depression and major depression in the National Comorbidity Survey. J Affect Disord.

[25] Weissman MM, Myers JK (1978). Affective disorders in a US urban community: the use of research diagnostic criteria in an epidemiological survey. Arch Gen Psychiatry.

[26] Meltzer H, Gill B, Petticrew M, Hinds K (1995). OPCS surveys of psychiatric morbidity in Great Britain, report 1: the prevalence of psychiatric morbidity among adults living in private households.

